# Transparent and Ultra-Thin Flexible Checkerboard Metasurface for Radar–Infrared Bi-Stealth

**DOI:** 10.3390/s24051531

**Published:** 2024-02-27

**Authors:** Qi Chang, Jinzu Ji, Ke Chen, Wenxing Wu, Yunpeng Ma

**Affiliations:** School of Aeronautic Science and Engineering, Beihang University, Beijing 100191, China; by2105303@buaa.edu.cn (Q.C.); jijinzu@buaa.edu.cn (J.J.); sy2305226@buaa.edu.cn (K.C.); by2305405@buaa.edu.cn (W.W.)

**Keywords:** checkerboard metasurface, radar–infrared bi-stealth, transparent, flexible

## Abstract

This paper proposed a single-layer checkerboard metasurface with simultaneous wideband radar cross-section (RCS) reduction characteristics and low infrared (IR) emissivity. The metasurface consists of an indium tin oxide (ITO)-patterned film, a polyethylene terephthalate (PET) substrate and an ITO backplane from the top downwards, with a total ultra-thin thickness of 1.6 mm. This design also allows the metasurface to have good optical transparency and flexibility. Based on phase cancellation and absorption, the metasurface can achieve a wideband RCS reduction of 10 dB from 10.6 to 19.4 GHz under normal incidence. When the metasurface is slightly cylindrically curved, an RCS reduction of approximately 10 dB can still be achieved from 11 to 19 GHz. The polarization and angular stability of the metasurface have also been verified. The filling rate of the top ITO-patterned film is 0.81, which makes the metasurface have a low theoretical IR emissivity of 0.24. Both simulation and experimental results have verified the excellent characteristics of the proposed checkerboard metasurface, demonstrating its great potential application in radar–IR bi-stealth.

## 1. Introduction

The development of modern radar detection technology has brought great challenges to the stealth characteristics of military targets. Radar cross-section (RCS) is an important indicator to measure the stealth capability of targets, and reducing RCS can effectively improve the survivability of targets [1,2]. Applying radar-absorbing materials (RAMs) and redirecting scattering fields are two commonly used methods for RCS reduction [3,4]. RAMs can convert incident energy to heat, and have been widely studied for their advantage of convenience and efficiency. Although some broadband RAMs have been proposed, they have the disadvantage of being too thick or too complex in structure [5,6]. Changing the shape of targets to keep the scattered field away from the direction is another feasible way to reduce RCS, but this may seriously affect the mechanical performance of targets.

Metasurfaces have the powerful ability to manipulate electromagnetic (EM) waves, which provides new strategies to achieve RCS reduction [7,8,9,10]. Through different design methods, metasurfaces can suppress scattering field through absorption, phase cancellation, and polarization conversion [11,12,13]. Among them, the checkerboard metasurface, based on phase interference cancellation technology, makes it easier to effectively reduce RCS in a wide frequency band, while avoiding thermal accumulation [14,15]. The main principle of a checkerboard metasurface is to use different types of artificial magnetic conductor (AMC) patterns and to make their reflection phase difference equal approximately 180°. The simplest structure is to use two types of AMC units with a reflection phase difference within $180^{\circ }\pm \;37^{\circ }$, and many structures based on this principle have been designed [16,17]. Y. Modi et al. designed a blended checkerboard metasurface using square rings and circular patch elements, which could achieve 10 dB RCS reduction in 3.9–9.45 GHz [18]. F. El-Sewedy et al. proposed a thin metasurface composed of triangular patches with different sizes, which had a thickness of only 0.8 mm but could achieve a 10 dB RCS reduction in 23.71–33.52 GHz [19]. Random and coded checkerboard metasurfaces have also been extensively studied, and through the algorithmic optimization of the layout, the frequency range of RCS reduction can be greatly expanded [20,21,22]. In [23], a binary-coded metasurface was designed, based on machine learning optimisation techniques, to achieve 10 dB RCS reduction in 14–20 GHz. Qu et al. proposed an optimized multi-element phase cancellation (OMEPC) method to design checkerboard metasurfaces [24]. By optimizing the shape and size of various unit cells, a 10 dB RCS reduction can be achieved in the ultra-wideband frequency band of 7.4–64.8 GHz.

With the increasing application of multiple detection, materials and structures with radar–IR bi-stealth are gradually attracting attention. Some composites with both microwave absorption and thermal insulation ability have been proposed to achieve radar–IR integrated stealth, such as Ni-MXene/Melamine [25], Fe/Fe_2_O_3_ porous carbon [26]. However, these composite materials are difficult to fabricate and have narrow absorption bands. Since metals usually have low IR emissivity, covering metamaterial absorbers with high filling rate metal patches is an effective method to achieve radar–IR bi-stealth, and many structures based on this method have been proposed [27,28,29,30,31]. It should be noted that covering metal patches will change the impedance matching of metamaterial absorbers, thus changing the original absorbing effect. For special applications such as aircraft windows and satellite solar panels, radar–IR bi-stealth metamaterial absorber with simultaneous optical transparency is designed [32,33,34]. However, metamaterial absorbers convert absorbed energy into thermal energy, which is not conducive to IR stealth. In addition, these structures are usually multi-layered, which suffers from the disadvantages of being too thick and less flexible. Some studies have proposed using checkerboard metasurface structures with high metal filling rates to achieve radar–IR stealth, but these structures have the disadvantages of large IR emissivity and thickness [35,36,37].

In this work, a single-layer checkerboard metasurface with simultaneous optical transparency, ultra-thin, flexible, wideband RCS reduction, and low IR emissivity is proposed. The proposed structure consists of a top indium tin oxide (ITO)-patterned film, a middle polyethylene terephthalate (PET) substrate, and an ITO backplane. The total structural thickness is only 1.6mm, which allows for good flexibility. The top ITO-patterned film contains two types of units with high ITO filling rates and, therefore, has lower IR emissivity. Finally, a sample of the proposed checkerboard metasurface was fabricated, and its RCS reduction performance and IR emissivity were verified through microwave darkroom RCS testing and thermal IR imaging.

## 2. Design and Analysis

**Figure 1:**
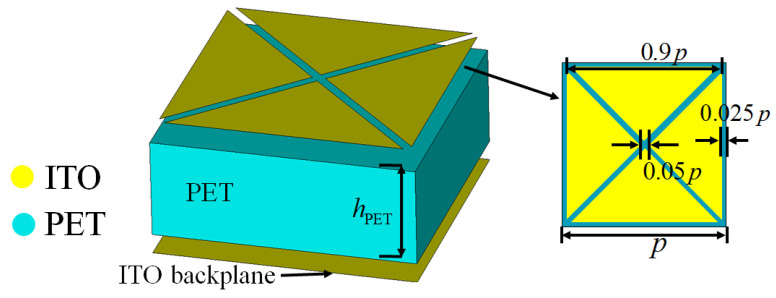
Unit cell design of the checkerboard metasurface.

In order to have good optical transparency and flexibility, the designed metasurface is only composed of a top ITO-patterned film, a PET substrate, and an ITO backplane. The IR radiation intensity of the metasurface can be calculated by the Stefan–Boltzmann equation as $j=\varepsilon \sigma T^{4}$, where ε is the IR emissivity, σ is the Stefan constant, and *T* is the temperature. As the temperature *T* of the metasurface is difficult to control, the IR radiation can be reduced by reducing ε. The IR emissivity $\varepsilon_{\mathrm{ITO}}$ of ITO increases with the increase of sheet resistance *R*. When *R* is less than 10 Ω/sq, $\varepsilon_{\mathrm{ITO}}$ is about 0.09 [31]. Since the operating wavelength range of the infrared band is only 3–12 μm, which is much lower than the radar wavelength, the IR characteristics of the metasurface mainly depend on the top ITO film pattern. The IR emissivity of PET is $\varepsilon_{\mathrm{PET}}=0.9$. Then, the total IR emissivity can be calculated as $\varepsilon_{\mathrm{TOTAL}}=\varepsilon_{\mathrm{ITO}}f_{\mathrm{ITO}}+\varepsilon_{\mathrm{PET}}\left(1-f_{\mathrm{ITO}}\right)$, where $f_{\mathrm{ITO}}$ is the filling rate of ITO. As the IR emissivity of PET is much larger than that of ITO, $\varepsilon_{\mathrm{TOTAL}}$ decreases with the increase of $f_{\mathrm{ITO}}$. But when $f_{\mathrm{ITO}}$ = one, the incident radar wave is almost fully reflected, which cannot achieve the effect of reducing RCS. Therefore, the top ITO pattern can be made into a bandpass periodic array with a high filling rate. Figure 1 shows the basic unit cell of the metasurface. The ITO pattern consists of four triangular patches with a sheet resistance of $R_{1}$ = 5 Ω/sq, and the period size is *p*. This design allows the pattern to have multiple gaps, which can generate more resonant modes. The relative permittivity of PET is approximately three, and its loss on the incident wave can be ignored [37]. It can be calculated that $f_{\mathrm{ITO}}$ and $\varepsilon_{\mathrm{TOTAL}}$ are 0.81 and 0.24, respectively.

The function of ITO backplane with a sheet resistance of $R_{2}$ is to reduce the transmittance of radar waves. It can be inferred that the radar wave transmittance decreases with the decrease of $R_{2}$. When $R_{2}$ is zero, the ITO backplane is equivalent to an perfect electric conductor (PEC) surface, and there will be no transmission at this time. The sheet resistance of ITO backplane is select as 5 Ω/sq, which can make the transmittance lower than −20 dB.

**Figure 2:**
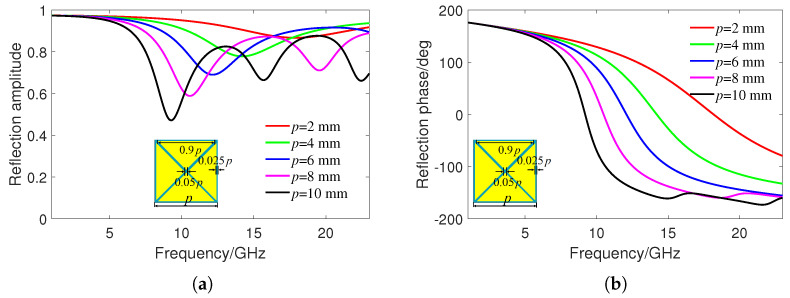
(**a**) Reflection amplitude and (**b**) reflection phase of basic units under different period sizes.

Figure 2 shows the reflection amplitude and phase of the basic unit at different period sizes *p*. The frequency range of the incident wave is 0–23 GHz, and the thickness of PET substrate is $h_{\mathrm{PET}}$ = 1.6 mm. These results were obtained using CST Studio Suite 2022 software. The frequency domain solver is adopted, where the “unit cell” boundaries were set in the xy plane and the "add open space" boundaries were set in the *z* direction. The reflection amplitude value is $S_{11}$. Due to the absorption effect of ITO, it can be seen that the reflectivity is not one. As the increase of *p*, the reflection amplitude curve generates more resonances, with the lowest reflection amplitude of 0.54 at *p* = 10 mm. Therefore, a 10 dB RCS reduction will not be achieved by relying on the absorption of one particular size of unit. From Figure 2b, it can be seen that there are significant differences in the reflection phase of units with different period sizes. Therefore, units of different sizes can be arranged in a checkerboard shape, and the principle of reflect phase cancellation can be used to achieve wideband RCS reduction.

**Figure 3:**
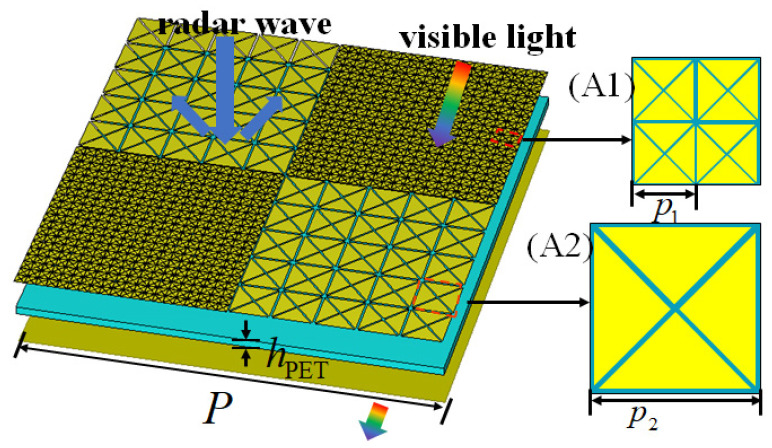
One period of the checkerboard metasurface and the schematic of RCS reduction, low IR emissivity, and optical transparency.

To make the metasurface have better polarization stability, two basic units of different sizes are used. Figure 3 shows a period of the proposed checkerboard metasurface, and also illustrates its working mechanism, which can scatter radar waves in multiple directions to reduce RCS in a specific direction, while having low IR emissivity and being able to penetrate visible light. The metasurface contains two different basic units A1 and A2, with periodic sizes of $p_{1}$ and $p_{2}$, respectively. For a checkerboard metasurface containing two types of units, an approximated expression for RCS reduction is presented as in [26], as follows:

(1) $${\mathrm{RCS}}_{\mathrm{Reduction}}=20\log_{10}\left[\frac{E_{r1}e^{j\varphi_{r1}}+E_{r2}e^{j\varphi_{r2}}}{2}\right]$$

where $E_{r1}$ and $E_{r2}$ are the reflected amplitudes of A1 and A2, while $\varphi_{r1}$ and $\varphi_{r2}$ are their phases, respectively. By substituting the relevant parameters into (1), the final optimized $p_{1}$ and $p_{2}$ are 2 mm and 8 mm, respectively. The total metasurface has a period length of *P* = 80 mm. The period size of A2 is four times that of A1, so the 4 × 4 size A1 unit array can also be regarded as a basic unit. A1 and A2 form 20 × 20 and 5 × 5 arrays respectively, and are distributed crosswise.

**Figure 4:**
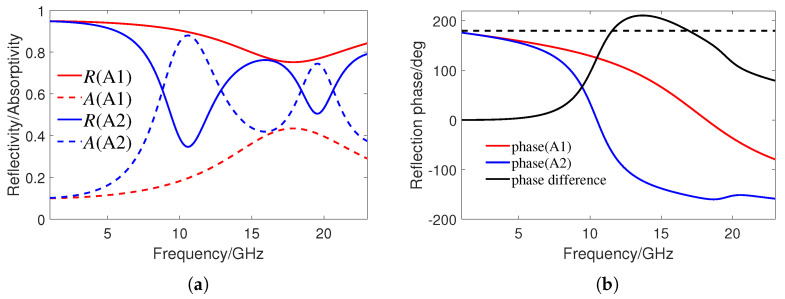
(**a**) Simulated reflectivity and absorptivity of A1 and A2. (**b**) Simulated reflection phases of A1, A2, and the phase difference between them. The vertical axis corresponding to the black dashed line is 180 °.

Figure 4a shows the reflectivity R(ω) and absorptivity A(ω) of the two units A1 and A2, while Figure 4b shows their corresponding reflection phases and their phase difference. The reflectivity is calculated by $R={\left|S_{11}\right|}^{2}$ and the absorptivity is A=1−R. It can be seen that both A1 and A2 have a certain absorption effect, where A1 generates a resonance point with a maximum absorption rate of 43% at 18.2 GHz, and A2 has two resonance points with a maximum absorption rate of 88% at 10.5 GHz. The phase difference between A1 and A2 at 11.6 GHz and 16.9 GHz is close to 180°, so it is expected to generate two reflection reduction peaks.

**Figure 5:**
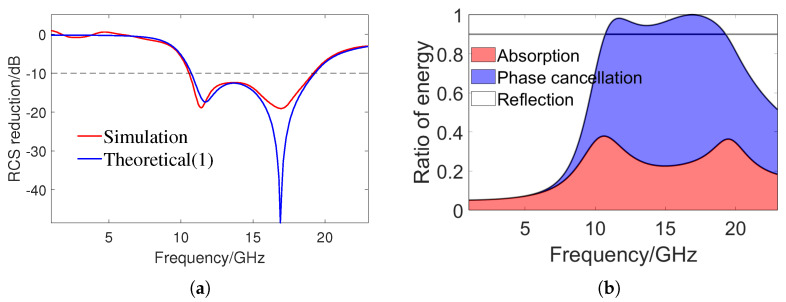
(**a**) RCS reduction obtained by full wave simulation and approximate RCS reduction obtained by (1) under normal incidence. (**b**) The energy distribution ratio of the metasurface, and the vertical axis corresponding to the black solid line is 0.9.

Under normal incidence, Figure 5a shows the RCS reduction obtained by a full-wave simulation and the approximate RCS reduction obtained by substituting the reflection coefficient and phase into (1). The full-wave simulation is calculated using the time domain solver of CST. The excitation source is a Gaussian pulse, and the RCS results in a wide frequency band are obtained through a time–frequency conversion. The time domain solver uses hexahedral meshes for spatial discretization, which cannot perfectly fit the geometric characteristics of the metasurface. Therefore, there are also certain errors in the simulation results. The metasurface is placed in the xy plane, and the angles between the incident wave direction with the *z*-axis and *x*-axis are θ and φ, respectively. The backscattering of the metasurface and the PEC surface with the same size is simulated, respectively, and the RCS reduction of the metasurface is obtained by subtracting the two results. It can be seen that the full-wave simulation results are basically consistent with the theoretical results obtained by (1). Considering the numerical error in the full-wave simulation, the difference between the two results is acceptable. The simulated RCS has a reduction of over 10 dB in the bandwidth of 10.6–19.4 GHz, with a relative bandwidth of 58.6%. As predicted, there are two RCS reduction peaks, reaching 18.9 dB and 19.1 dB at 11.8 GHz and 16.8 GHz, respectively. Figure 5b shows the energy distribution ratio of the proposed checkerboard metasurface under normal incidence, where the energy ratio of absorption A(ω), phase cancellation PC(ω), and reflection R(ω) are calculated as follows:

(2) $$\begin{array}{cc} \; & A\left(\omega \right)=\sum_{i=1}^{2}(1-A_{i}^{2})/2\\ \; & PC\left(\omega \right)={\left|\sum_{i=1}^{2}A_{i}e^{j\phi_{i}}/2\right|}^{2}\\ \; & R(\omega )=1-A(\omega )-PC(\omega ) \end{array}$$

When the total energy ratio of the absorption and phase interference exceeds 90%, the reflected energy ratio will be less than 10%, and the RCS reduction will exceed 10 dB. It can be concluded that the achievement of broadband RCS reduction is the result of the combined effect of absorption and phase cancellation, and the contribution of phase cancellation to RCS reduction is greater than that of absorption.

**Figure 6:**
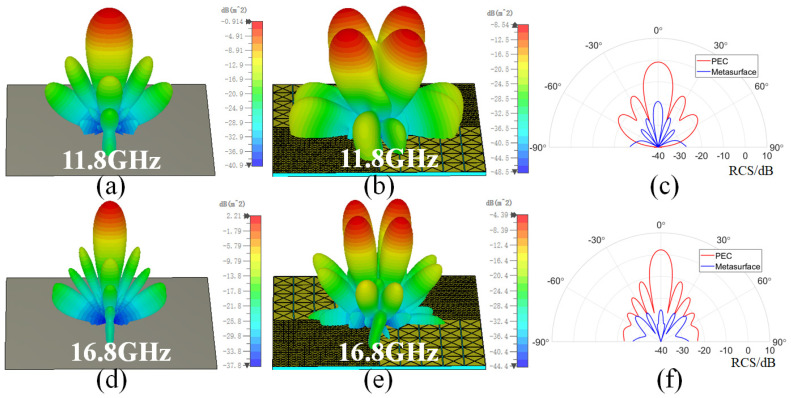
The 3D scattering patterns of the PEC surface and the checkerboard metasurface, as well as the corresponding bistatic RCS in the xz plane at different frequencies. (**a**–**c**) 11.8 GHz. (**d**–**f**) 16.8 GHz.

Figure 6 shows the three-dimensional (3D) scattering patterns of the checkerboard metasurface and equal-sized PEC surface at frequencies of 11.8 and 16.8 GHz under normal incidence, as well as the corresponding bistatic RCS in the xz plane. Due to the symmetry of the metasurface, the scattering patterns at this time are not affected by the polarization mode. It can be seen that the main scattering lobe of the PEC surface is concentrated in the normal incident direction. Four scattering lobes appear on the metasurface, and the scattering intensity is much lower than that of PEC surface. The bistatic RCS of the metasurface in the xz plane is lower than that of the PEC surface. In the direction of $\theta \;=\;0^{\circ }$, there is a significant decrease in the scattering intensity of the metasurface. The backscattering RCS of the PEC surface and the metasurface at 11.8 GHz are −0.9 dB and −19 dB, and the backscattering RCS of the PEC surface and metasurface at 16.8 GHz are 2.2 dB and −25 dB. These results indicate that the metasurface has good RCS reduction performance at normal incidence.

**Figure 7:**
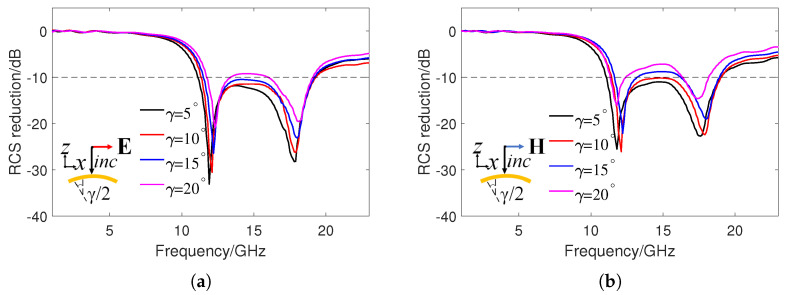
Simulated RCS reduction of the checkerboard metasurface under different curvature angles γ and polarizations. (**a**) TM polarization. (**b**) TE polarization.

**Figure 8:**
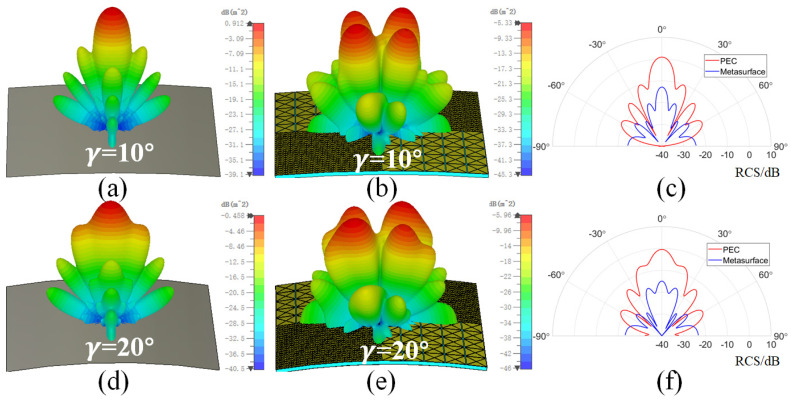
The 3D scattering patterns of the PEC surface and the checkerboard metasurface, as well as the corresponding bistatic RCS in the xz plane at different curvature angles. (**a**–**c**) γ = $10^{\circ }$. (**d**–**f**) γ = $20^{\circ }$.

Since the metasurface has an excellent flexibility, it can be cylindrically curved with a curvature angle of γ. Figure 7 shows the RCS reduction of the metasurface under different polarizations with γ values of $5^{\circ }$, $10^{\circ }$, $15^{\circ }$, and $20^{\circ }$ at 15 GHz. It can be seen that for TM and TE polarization, as the curvature angles γ increases, the RCS reduction effect gradually decreases. When γ is less than $15^{\circ }$, an RCS reduction of approximately 10 dB can still be achieved in the frequency range of 11–19 GHz. This is because the curvature changes the impedance match of the base unit, thereby changing the reflection amplitude and phase. For TM polarization, 10 dB RCS reduction can be achieved in a wide frequency band when γ is less than $15^{\circ }$, which has a better performance than TE polarization. This is because, under TM polarization, the surface current induced by the magnetic field is still parallel to the metasurface, and a more stable scattering pattern is generated [34]. Figure 8 shows the 3D scattering patterns of the PEC surface and the checkerboard metasurface with γ values of $10^{\circ }$ and $20^{\circ }$, as well as the bistatic RCS in the xz plane. The incident frequency is 15 GHz and the polarization mode is TM polarization. It can be seen that the main scattering lobe of the PEC surface is still concentrated in the normal incident direction, and four scattering lobes appear on the metasurface. In addition, it can be seen that the main scattering lobes have a wider range of azimuths when γ = $20^{\circ }$ than when γ = $10^{\circ }$. At this time, the bistatic RCS of the metasurface in the xz plane is still lower than the PEC surface. The backscattering RCS of the PEC surface and metasurface at γ = $10^{\circ }$ are 0.9 dB and −12.8 dB, and the backscattering RCS at γ = $20^{\circ }$ are −0.4 dB and −25 dB. Therefore, the designed checkerboard metasurface still has excellent RCS reduction performance after proper bending.

**Figure 9:**
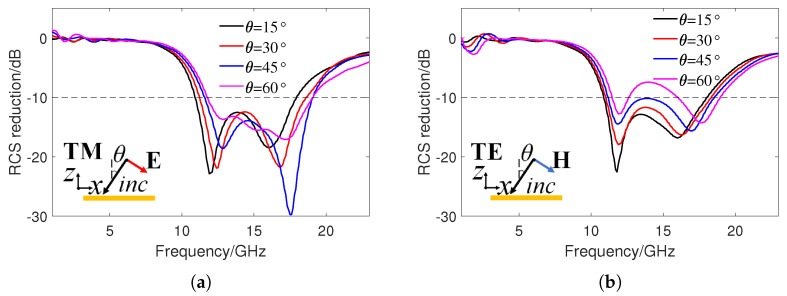
Simulated RCS reduction in specular direction of the checkerboard metasurface under different incident angles θ and polarizations. (**a**) TM polarization. (**b**) TE polarization.

**Figure 10:**
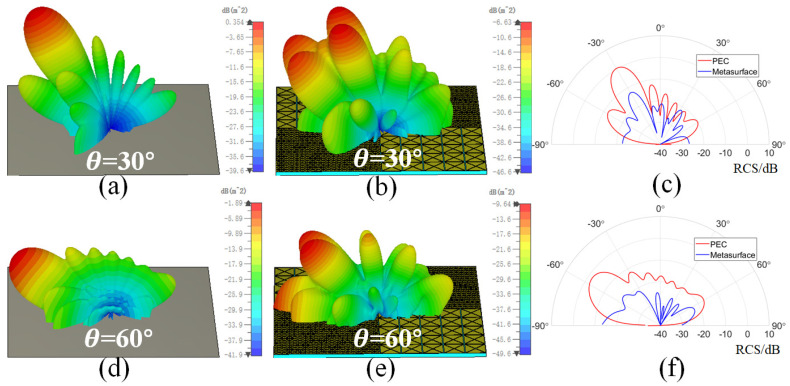
The 3D scattering patterns of the PEC surface and the checkerboard metasurface, as well as the corresponding bistatic RCS in the xz plane at different oblique incidence angles. (**a**–**c**) θ = $30^{\circ }$. (**d**–**f**) θ = $60^{\circ }$.

The RCS reduction effect of the checkerboard metasurface at different oblique incidence angles are also studied. When electromagnetic waves are obliquely incident on the PEC surface, the reflected lobes will mainly concentrate in the specular direction, so the RCS reduction in the specular direction is calculated here. Under oblique incidence, the surface impedance and phase difference of A1 and A2 are functions of the incidence angle. Figure 9 shows the RCS reduction results under different polarizations at incidence angles θ of $15^{\circ }$, $30^{\circ }$, $45^{\circ }$, and $60^{\circ }$. It can be seen that, when θ is less than 45°, the checkerboard metasurface can still achieve 10 dB RCS reduction in a wide frequency band under TE and TM polarization. For TM polarization, broadband 10 dB RCS reduction can still be achieved when θ is greater than $45^{\circ }$. However, for TE polarization, the RCS reduction performance deteriorates sharply when θ is greater than $45^{\circ }$. This is also because, under TM polarization, the magnetic field is parallel to the metasurface and, thus, generates a sufficiently large induced current, which is more conducive to the absorption of the incident wave. Also, for TE polarization, the magnetic field component parallel to the metasurface decreases with an increasing incident angle, ultimately leading to a decrease in absorption and RCS reduction effects. Figure 10 shows the 3D scattering patterns of PEC surface and the checkerboard metasurface with θ values of $30^{\circ }$ and $60^{\circ }$, as well as the bistatic RCS in the xz plane. The incident frequency is 15 GHz and the polarization mode is TM polarization. It can also be seen that the PEC surface generates one main scattering lobe in the specular direction, while the metasurface exhibits four scattering lobes. Compared to the PEC surface, the bistatic RCS of the metasurface in the xz plane is also significantly reduced.

## 3. Experimental Validation

**Figure 11:**
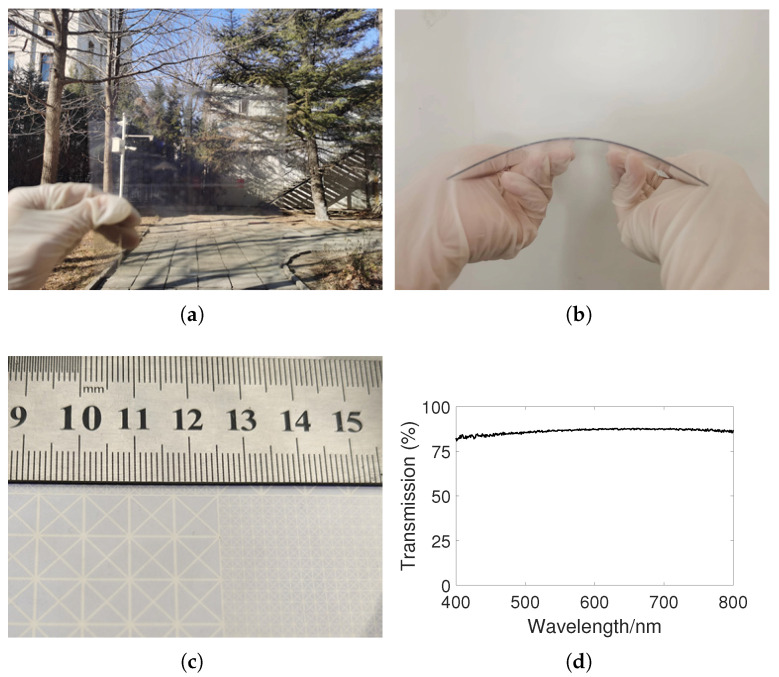
(**a**) Photograph of the fabricated sample showing optical transparency and (**b**) photograph of the fabricated sample showing flexibility. (**c**) Magnified photograph image of the fabricated sample. (**d**) The transmittance of the sample measured in the visible light wavelength range.

A sample of the metasurface consisting of a 2 × 2 period was fabricated and measured. First, a 180 nm thick ITO coating was deposited on a 0.05 mm thick PET film by magnetron sputtering technology, to obtain an ITO–PET film, which can be directly used as the ITO backplane of the metasurface. The ITO coating thickness had little effect on the total thickness of the film. Then, the ITO–PET film obtained in the previous step was processed using laser etching technology to obtain the ITO pattern on the top layer of the metasurface. The actual processed ITO film resistance is approximately 4–6 Ω/sq. The surface current of ITO pattern and the charge accumulation on the edge will generate inductive reactance and capacitive reactance, respectively. According to the method in [38], it can be calculated that the inductive reactance and capacitive reactance of the ITO pattern are much larger than the surface resistance, so the ITO film resistance error has little impact on the results. In addition, the full wave simulation results also verified that the difference in surface resistance had almost no impact on the results. Finally, we used transparent adhesive to stick the upper and lower layers of ITO–PET film onto a 1.5 mm thick PET substrate, to obtain the sample. From Figure 11a, the scene behind can be clearly seen through the sample, which verifies the good optical transparency of the checkerboard metasurface. The sample has good flexibility and can be easily cylindrically curved, as shown in Figure 11b. Figure 11c shows the magnified photograph image of the fabricated sample. The transparency of the metasurface in the visible wavelength range of 400–800 nm is measured using a ultraviolet (UV)/visible (Vis)/near-infrared (NIR) spectrometer, and Figure 11d shows the measurement results. Due to its ultra-thin thickness of 1.6 mm, the sample exhibits a transmittance of over 80% in the visible light band.

**Figure 12:**
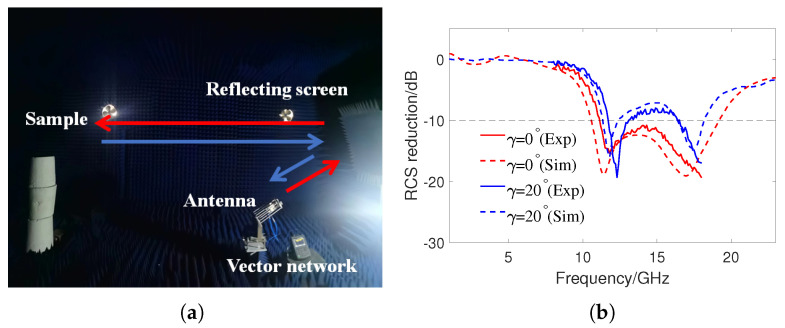
(**a**) Measurement setup in the anechoic chamber. (**b**) Simulated and measured RCS reduction at $\gamma \;=\;0^{\circ }$ and $20^{\circ }$ under normal incidence.

Figure 12a shows the experimental set up for RCS measurement in a microwave darkroom, in which two standard horn antennas are connected to a vector network analyzer (N9918A), and the operating frequency range of antennas is 8–18 GHz. The transmitting antenna emits signals and converts them into plane waves through a metal reflective screen. The scattered waves are reflected by the metal screen and received by the receiving antenna. Due to limitations in experimental conditions, only two cases under TE polarization at normal incidence were tested: when the metasurface was not curved and the metasurface has a cylindrically curved angle of $\gamma =20^{\circ }$. Figure 12b shows the comparison of RCS reduction results obtained from experimental testing and simulation for the two cases. It can be seen that, although there are differences between the experimental results and the simulation results, the overall trend of change is basically consistent. The reason for the difference is the inability to precisely control the orientation and curvature angle of the metasurface. In addition, there are numerical errors in the full-wave simulation results.

**Figure 13:**
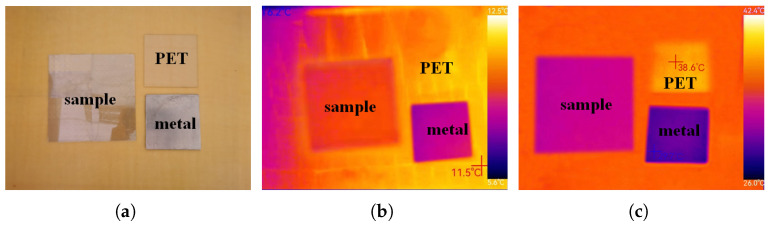
(**a**) Image of the checkerboard metasurface, metal, and PET plate. (**b**) Thermal IR image at 25 °C. (**c**) Thermal IR image at 50 °C.

The IR stealth ability of the proposed metasurface was tested through IR imaging. We imaged the fabricated sample using a thermal IR camera (Thermoview T72) (Huashengchang, Shenzhen, China) operating at the wavelength of 7.5–14 μm and compared the results with the IR imaging of a metal plate and a PET plate. Figure 13a shows the image of the three samples. Figure 13b is the IR image at a room temperature of 25 °C, and Figure 13c is the IR image of the three samples after being heated on a 50 °C heating plate for ten minutes. The higher the temperature displayed in the IR image, the stronger the IR radiation of the object. It can be found that the IR radiation of the checkerboard metasurface is higher than the metal plate, but is significantly lower than PET, which confirms the good IR stealth performance of the metasurface. Based on the imaging results, it can be estimated that the IR emissivity of the checkerboard metasurface is as follows [39]:

(3) $$\epsilon_{exp}=\left(T_{r}^{4}-T_{a}^{4}\right)/\left(T_{o}^{4}-T_{a}^{4}\right)$$

where $T_{r}$ is the temperature displayed in the IR image, $T_{o}$ is the ambient temperature of the metasurface, and $T_{a}$ is the atmospheric temperature. The ambient temperature is 50 °C, the atmospheric temperature is 25 °C, and the checkerboard metasurface in Figure 13c shows a temperature of about 32 °C. These three temperatures need to be converted into Kelvin temperatures when substituting into (3), and the result can be eventually calculated as $\epsilon_{exp}$ = 0.256. This is very close to the theoretical result, proving that the metasurface has an extremely low IR emissivity.

Table 1 shows a comprehensive comparison of the proposed checkerboard metasurface with some previously reported structures. The comparison shows that although the proposed metasurface has a relatively low 10 dB RCS reduction bandwidth, it has low thickness, optical transparency, low infrared emissivity, and flexibility at the same time. The thickness of the metasurface is only 0.08$\lambda_{L}$, where $\lambda_{L}$ is the wavelength at the center frequency of the 10 dB RCS reduction band. Therefore, compared with previously published radar–IR bi-stealth structures, the proposed metasurface has significant advantages in thickness and flexibility.

## 4. Conclusions

In summary, a checkerboard metasurface with simultaneous optical transparency, ultra-thin, flexible, wideband RCS reduction, and low IR emissivity is designed. Based on the hybrid mechanism of phase cancellation and absorption, the designed metasurface can effectively achieve 10 dB RCS reduction in the frequency band of 10.6–19.4 GHz, and has a low IR emissivity of 0.24. The total thickness of the metasurface is only 1.6 mm, which allows for good flexibility. The metasurface can still achieve a good RCS reduction effect under normal incidence when it is cylindrically curved. A sample of the checkerboard metasurface was fabricated and measured, and the experimental results also verified its excellent performance. The proposed checkerboard metasurface shows a good radar–IR bi-stealth capability, and has great potential for applications such as aircraft canopies and stealth glass.

## Figures and Tables

**Table 1:** A comprehensive comparison of the proposed checkerboard metasurface with some previously reported structures.

Ref.	10 dB RCS Reduction Range (GHz)/(RBW) ^a^	Thickness (mm)/${\left(\lambda_{L}\right)}^{b}$	Opt. Trans.	IR Emissivity	Flexibility	Working Mechanism
Ref. [18]	3.9–9.45/83%	6.35/0.14	No	/	No	Scattering
Ref. [19]	23.7–33.5/34%	0.8/0.07	No	/	No	Scattering
Ref. [24]	7.3–64.8/161%	5.9/0.74	No	/	No	Scattering
Ref. [34]	7.7–18/80%	4/0.17	Yes	0.24	Yes	Absorption
Ref. [35]	7.4–13.4/57%	3.35/0.12	Yes	0.31	No	Hybrid
Ref. [36]	8.6–16.3/62%	4.4/0.18	No	0.43	Yes	Hybrid
Ref. [37]	8.2–16.0/64%	2.26/0.09	No	0.2	No	Absorption
This work	10.6–19.4/58.6%	1.6/0.08	Yes	0.24	Yes	Hybrid

^a^ Relative bandwidth; ^b^ the wavelength corresponding to the center frequency of the 10 dB RCS reduction band.

## Data Availability

The data that support the findings of this study are available from the corresponding author upon reasonable request.

## References

[1] Heidari M., Sedighy S.H., Amirhosseini M.K. (2023). RCS reduction using grounded multi-height multi-dielectrics metasurfaces. Sci. Rep..

[2] Wang C., Wang R.Z., Zhang S.J., Wang H., Wang W.S. (2023). Reducing radar cross section of flat metallic targets using checkerboard metasurface: Design, analysis, and realization. J. Appl. Phys..

[3] Li W., Xu M., Xu H.X., Wang X., Huang W. (2022). Metamaterial Absorbers: From Tunable Surface to Structural Transformation. Adv. Mater..

[4] Wang B., Xu C., Duan G., Xu W., Pi F. (2023). Review of Broadband Metamaterial Absorbers: From Principles, Design Strategies, and Tunable Properties to Functional Applications. Adv. Funct. Mater..

[5] Lee C., Kim K., Park P., Jang Y., Jo J., Choi T., Lee H. (2023). Ultra-Wideband Electromagnetic Composite Absorber Based on Pixelated Metasurface with Optimization Algorithm. Materials.

[6] Takeshita H., Nita D., Cheng Y., Fathnan A.A., Wakatsuchi H. (2023). Dual-band waveform-selective metasurfaces for reflection suppression. Appl. Phys. Lett..

[7] Chang Q., Ma Y., Liu J., Wu W., Ji X., Ji J. (2022). Equivalent circuit model for reflective polarization converter based on anisotropic metasurfaces. J. Appl. Phys..

[8] Min P., Song Z., Yang L., Ralchenko V.G., Zhu J. (2022). Multispectral meta-film design: Simultaneous realization of wideband microwave absorption, low infrared emissivity, and visible transparency. Opt. Express.

[9] Ju Z., Wen J., Shi L., Yu B., Deng M., Zhang D., Hao W., Wang J., Chen S., Chen L. (2020). Ultra-Broadband High-Efficiency Airy Optical Beams Generated with All-Silicon Metasurfaces. Adv. Opt. Mater..

[10] Cheng Y.Z., Huang M.L., Chen H.R., Guo Z.Z., Mao X.S., Gong R.Z. (2017). Ultrathin Six-Band Polarization-Insensitive Perfect Metamaterial Absorber Based on a Cross-Cave Patch Resonator for Terahertz Waves. Materials.

[11] Li F., You B. (2022). Complementary Multi-Band Dual Polarization Conversion Metasurface and Its RCS Reduction Application. Electronics.

[12] Huang Z., Zheng Y., Li J., Cheng Y., Wang J., Zhou Z.K., Chen L. (2023). High-Resolution Metalens Imaging Polarimetry. Nano Lett..

[13] Goncalves Licursi de Mello R., Lepage A.C., Begaud X. (2023). A Low-Profile, Triple-Band, and Wideband Antenna Using Dual-Band AMC. Sensors.

[14] Fang S., Deng L., Zhang P., Qiu L., Xie H., Huang S., Du J., Wang Z. (2022). Dual-function flexible metasurface for absorption and polarization conversion and its application for radar cross section reduction. J. Appl. Phys..

[15] Chen W., Balanis C.A., Birtcher C.R. (2015). Checkerboard EBG Surfaces for Wideband Radar Cross Section Reduction. IEEE Trans. Antennas Propag..

[16] Zhou C.F., Yu Q.F., Gustafson C., Lau B.K. (2023). Wideband RCS reduction based on a simple chessboard metasurface. J. Appl. Phys..

[17] Khan T.A., Li J., Chen J., Raza M.U., Zhang A. (2019). Design of a Low Scattering Metasurface for Stealth Applications. Materials.

[18] Modi A.Y., Balanis C.A., Birtcher C.R., Shaman H.N. (2017). Novel Design of Ultrabroadband Radar Cross Section Reduction Surfaces Using Artificial Magnetic Conductors. IEEE Trans. Antennas Propag..

[19] El-Sewedy M.F., Abdalla M.A. (2023). A Monostatic and Bistatic RCS Reduction Using Artificial Magnetic Conductor Metasurface. IEEE Trans. Antennas Propag..

[20] Moccia M., Liu S., Wu R.Y., Castaldi G., Andreone A., Cui T.J., Galdi V. (2017). Coding Metasurfaces for Diffuse Scattering: Scaling Laws, Bounds, and Suboptimal Design. Adv. Opt. Mater..

[21] Su J., Lu Y., Liu J., Yang Y., Li Z., Song J. (2018). A Novel Checkerboard Metasurface Based on Optimized Multielement Phase Cancellation for Superwideband RCS Reduction. IEEE Trans. Antennas Propag..

[22] Zhao Z., Li X., Dong G. (2023). Wideband RCS Reduction Based on Hybrid Checkerboard Metasurface. Sensors.

[23] Chittur Subramanianprasad P., Ma Y., Ihalage A.A., Hao Y. (2023). Active Learning Optimisation of Binary Coded Metasurface Consisting of Wideband Meta-Atoms. Sensors.

[24] Qu M., Zhang C., Su J., Liu J., Li Z. (2022). Extremely Wideband and Omnidirectional RCS Reduction for Wide-Angle Oblique Incidence. IEEE Trans. Antennas Propag..

[25] Cheng H., Pan Y., Wang X., Liu C., Shen C., Schubert D.W., Guo Z., Liu X. (2022). Ni Flower/MXene-Melamine Foam Derived 3D Magnetic/Conductive Networks for Ultra-Efficient Microwave Absorption and Infrared Stealth. Nanomicro Lett..

[26] Zhu X., Dong Y., Xiang Z., Cai L., Pan F., Zhang X., Shi Z., Lu W. (2021). Morphology-controllable synthesis of polyurethane-derived highly cross-linked 3D networks for multifunctional and efficient electromagnetic wave absorption. Carbon.

[27] Zhong S., Jiang W., Xu P., Liu T., Huang J., Ma Y. (2017). A radar-infrared bi-stealth structure based on metasurfaces. Appl. Phys. Lett..

[28] Kim T., Bae J., Lee N., Cho H.H. (2019). Hierarchical Metamaterials for Multispectral Camouflage of Infrared and Microwaves. Adv. Funct. Mater..

[29] Wang L., Dong J., Zhang W., Zheng C., Liu L. (2023). Deep Learning Assisted Optimization of Metasurface for Multi-Band Compatible Infrared Stealth and Radiative Thermal Management. Nanomaterials.

[30] Pang Y., Li Y., Yan M., Liu D., Wang J., Xu Z., Qu S. (2018). Hybrid metasurfaces for microwave reflection and infrared emission reduction. Opt. Express.

[31] Chang Q., Wu W., Ma Y., Ji X., Ji J. (2023). Transparent and tunable water-based metamaterial absorber with low infrared emissivity. Appl. Phys. Lett..

[32] Chang Q., Ji J., Wu W., Ma Y. (2023). An Optically Transparent Metamaterial Absorber with Tunable Absorption Bandwidth and Low Infrared Emissivity. Materials.

[33] Lee N., Lim J., Chang I., Bae H.M., Nam J., Cho H.H. (2022). Flexible Assembled Metamaterials for Infrared and Microwave Camouflage. Adv. Opt. Mater..

[34] Zhang C., Wu X., Huang C., Peng J., Ji C., Yang J., Huang Y., Guo Y., Luo X. (2019). Flexible and Transparent Microwave–Infrared Bistealth Structure. Adv. Mater. Technol..

[35] Wang Y., Wu G., Wang Y., Jia Q., Liu J. (2023). Single-layer metasurface: Optical transparency, microwave scattering reduction and infrared emissivity decrease. Opt. Mater..

[36] Zhang W., Xu C., Cheng A., Fan Q., Wang J., Qu S. (2022). Infrared stealth and the flexible metasurface of radar backscattering suppression. J. Phys. D Appl. Phys..

[37] Zhang C., Yang J., Yuan W., Zhao J., Dai J.Y., Guo T.C., Liang J., Xu G.Y., Cheng Q., Cui T.J. (2017). An ultralight and thin metasurface for radar-infrared bi-stealth applications. J. Phys. D Appl. Phys..

[38] Costa F., Monorchio A., Manara G. (2010). Analysis and Design of Ultra Thin Electromagnetic Absorbers Comprising Resistively Loaded High Impedance Surfaces. IEEE Trans. Antennas Propag..

[39] Jing H., Wei Y., Kang J., Song C., Deng H., Duan J., Qu Z., Wang J., Zhang B. (2023). An optically transparent flexible metasurface absorber with broadband radar absorption and low infrared emissivity. J. Phys. D Appl. Phys..

